# DANTE-SPACE: a new technical tool for DVT on 1.5T MRI

**DOI:** 10.1007/s10554-019-01675-w

**Published:** 2019-08-24

**Authors:** Gaoming Zhuang, Caiyun Tang, Xueping He, Jianke Liang, Zhuonan He, Yufeng Ye, Wei Deng, Dexiang Liu, Hanwei Chen

**Affiliations:** 1 The First Affiliated Hospital, Jinan University, Guangzhou, Guangdong China; 2 Department of Radiology, Guangzhou Panyu Central Hospital, Guangzhou, 511400 Guangdong China; 3 Medical Imaging Institute of Panyu, Guangzhou Panyu Central Hospital, Guangzhou, 511400 Guangdong China

**Keywords:** Magnetic resonance imaging (MRI), DANTE-SPACE, Deep venous thrombosis (DVT), Contrast-enhanced magnetic resonance venography (CE-MRV), Gadolinium

## Abstract

The aim of the study was to compare CE-MRV with DANTE-SPACE on a 1.5T MRI system for the diagnosis of DVT. The patients were diagnosed with deep venous thrombosis of the lower extremities based on swelling, pain, and superficial varicose veins of the lower extremities. MRI examination confirmed the diagnosis. DANTE-SPACE images were obtained before the conventional contrast-enhanced MRV, which uses gadolinium. The scanning field started from the end of the inferior vena cava to the end of the ankle, divided into five observation segments, namely, the common iliac vein, external iliac vein, femoral vein, popliteal vein, and calf vein. The DANTE-SPACE and CE-MRV results were used for a consistency analysis. For the DANTE-SPACE and CE-MRV images, the signal intensity ratios of the thrombus/cavity and thrombus/muscle were calculated, and the ratio difference was compared using the paired *t* test. Twenty-six patients completed the examination; one of the patients underwent a right lower limb amputation, yielding a total of 255 lower limb vascular segments. The analysis of the DANTE-SPACE images showed that there were 14 iliac vein thromboses, 18 external iliac vein thromboses, 23 femoral vein thrombi, 21 popliteal vein thromboses, and 18 calf vein thromboses; these findings were consistent with the diagnostic results of CE-MRV. The ratio of the thrombus/cavity signal intensity measured in the DANTE-SPACE and CE-MRV images were as follows: 20.51 ± 12.96 vs. 0.51 ± 0.46; P < 0.05, n = 51; the difference was statistically significant. The ratio of the thrombus/muscle signal intensity measured on the DANTE-SPACE and CE-MRV images were as follows: 1.74 ± 0.57 vs. 0.99 ± 0.53; P < 0.05, n = 51; the difference was statistically significant. Compared with CE-MRV, DANTE-SPACE showed no significant difference in the ability to detect deep venous thrombosis of the lower extremities. DANTE-SPACE did not use contrast-enhancing agents and showed no evidence of inflammatory enhancement, and the display effect of small diameter veins was slightly poor. However, deep venous thrombosis of the lower extremities presents different levels of high signal in the DANTE-SPACE images, making it easy to identify and diagnose. It can also indicate the different components and age of the thrombus and help with the selection of a more accurate clinical treatment plan. MRI DANTE-SPACE is the preferred imaging modality for patients with deep venous thrombosis who are unable or unwilling to use gadolinium contrast agents.

## Introduction

Deep venous thrombosis (DVT) is a venous blood flow disorder caused by the abnormal coagulation of blood in the deep veins of the lower extremities, and it is one of the most common peripheral vascular diseases seen in the outpatient clinic. DVT may undergo thrombus shedding, causing pulmonary embolism (PE) and possibly life-threatening venous thromboembolism (VTE). If the thrombus has not shed and is not treated promptly, patients with DVT can develop post-thrombotic syndrome (PTS), causing long-term pain that severely affects their life, mobility, and ability to work. There are therefore many clinical studies in progress on the diagnosis and treatment of DVT.

Ultrasonography, which is inexpensive and noninvasive, is the primary diagnostic method for DVT. The sensitivity and specificity of ultrasonography for DVT are both more than 95% [1]. However, the method still has some limitations. It is not reliable for the examination of the common iliac vein and internal iliac vein, and it is inefficient for the evaluation of the duration of thrombus formation or for its internal structure. With the progress of DVT treatment technology, particularly the popularity of mechanical thrombectomy and thrombolysis, the shortcomings of ultrasonography are becoming more obvious.

Contrast-enhancement magnetic resonance venography (CE-MRV), which is increasingly being used to diagnose DVT, is noninvasive, reproducible, and has the advantage of the use of non-ionizing radiation. A study on CE-MRV concluded that the sensitivity and specificity for acute DVT were 91.0% and 99.8%, respectively, and were 84.4% and 98.4%, respectively, for chronic DVT [2]. However, CE-MRV needs to accurately estimate the cycle time of the target blood vessel, and the scanning operation is difficult. Simultaneously, the use of contrast agents not only increases the examination costs but also increases the risk of allergy and limitations, because the contrast agents cannot be used in patients with severe renal insufficiency or in pregnant women. The use of non-enhanced MRI technology to diagnose DVT gradually increased over the years as a result.

The Delay Alternating with Nutation for Tailored Excitation; Sampling Perfection with Application-optimized Contrasts by using different flip angle Evolutions (DANTE-SPACE) is a 3D black-blood thrombus imaging (BTI) technique. Some studies suggest that it is superior to SPACE and MPRAGE and may be the ideal technique for the noninvasive diagnosis of DVT [3]. The purpose of this study was to compare CE-MRV with DANTE-SPACE on a 1.5T MRI system for the diagnosis of DVT.

## Materials and methods

The patients were hospitalized from September 2016 to June 2017. The symptoms of DVT included swelling of the lower extremities, superficial varicose veins, and pain in the lower extremities. After the initial clinical examination, a suspected DVT had to be confirmed with CE-MRI. Patient consent to add non-enhanced DANTE-SPACE sequences to conventional CE-MRV scans was obtained before the examination. After the completion of MRI examination, 7 patients (7/26) received interventional thrombolytic therapy in our hospital, and 19 patients chose other treatment methods. After case tracking, we obtained the DSA data of the lower limb deep vein angiography of these 7 patients. (The experiment was approved by the author's hospital Institutional Review Board (IRB). The number is H20170024.)

The inclusion criteria for the study were: (1) Patients with DVT symptoms or signs, swelling of the lower extremities, superficial varices, lower extremity pain, etc. (2) MRI examination was completed within 7 days after the initial clinical diagnosis, and treatment for DVT was not accepted. (3)18 years old or older, conscious, informed consent. (4) No CE-MRI examination contraindications. The exclusion criteria for the study were: (1) contraindications for MRI Examination, contrast allergies, renal insufficiency (glomerular filtration rate < 30 ml/min), claustrophobia. (2) History of surgery within 48 h prior to MRI. (3) Reluctant to participate in the research project.

The patients were scanned with a Magnetom Avanto1.5T magnetic resonance scanner (Siemens AG, Germany) and a body-dedicated phased array coil. The contrast agent used was Magnevist injection (gadopentetate dimeglumine, Bayer, Germany), 0.5 mmol/mL, and 20 ml vial. The scanning methods were as follows: (1) positioning for scanning included the transverse, coronal, and sagittal positions; (2) routine scan with the SE T1WI, and FSE T2WI sequence was conducted; (3) DANTE-SPACE: a repeat of the three lower limb coverage scanning was conducted; (4) a total of 30 ml of contrast agent, with a 20 ml saline injection washout, was injected into the elbow vein at a speed of 3 ml/s; (5) using real-time imaging monitoring techniques, the arterial imaging was noted to be immediately triggering the arterial phase scan, and the venous scan was initiated 30 s later; (6) automatic bed removal technology scanning was conducted; and (7) DANTE-SPACE and CE-MRV scan data were automatically spliced, and parallel MIP and MPR post-processing were conducted to optimize the images.

The DANTE-SPACE parameters were as follows: TR = 660 ms; TE = 11 ms; flip angle (°) T1 variable; Interpolated voxel size (mm^3^) 0.7 × 0.7 × 0.7; scan field = 300 mm × 400 mm; DANTE pulse train length 125; DANTE flip angle 15 degrees; section scan time = 240 s; and time needed to cover the lower extremity = 720 s. The CE-MRV parameters were as follows: VIBE + FS TR = 3.89 mms; TE = 1.42 mms; scan field = 300 mm × 400 mm; flip angle = 25 degrees; section scan time = 19 s; and time needed to cover the lower extremity = 57 s.

For the image analysis and measurement, unilateral lower extremity veins were divided into five observation sections, namely, the common iliac vein, external iliac vein, femoral vein, popliteal vein, and calf vein. The distribution of the thrombus was recorded in the DANTE-SPACE and CE-MRA images. In the DANTE-SPACE images, the MRI signals at the brightest point of the thrombosis were measured. The MRI signal value of the muscle was measured at the homogenous adjacent signal. The MRI signal value of the proximal normal venous cavity was measured, and the MRI signal value was measured at the contralateral normal vena cava when the venous thrombosis was widespread in the unilateral lower extremity vein. In the CE-MRV images, the thicker, clearer, and more uniform thrombus was chosen to allow measurement of the MRI signal value; that of the muscle was measured where the adjacent signal was uniform. The MRI signal value in the proximal normal venous cavity of the thrombus was measured. When the lower extremity vein was extensively obstructed, the MRI signal value was measured in the contralateral normal venous cavity. Image observation and data measurement were performed by two physicians with more than 5 years of DVT diagnosis experience. The analysis was blind for the clinical status. Each physician needs to diagnose whether there is thrombosis in each segment of vein. When the diagnosis results are inconsistent, consensus is reached through consultation. The two physicians discussed and determined the data measurement points. Each measurement point was measured three times and its average value was taken.

The IBM SPSS19.0 software package was used for the data analysis. The qualitative diagnosis of DVT obtained from DANTE-SPACE and CE-MRV was performed using the *Kappa* consistency test and paired *χ*^2^ test (McNemar test)*.* The quantitative analysis of the ratios of thrombus/cavity and thrombus/muscle was performed using the Wilcoxon Rank Sum Test.

## Results

**Fig. 1:**
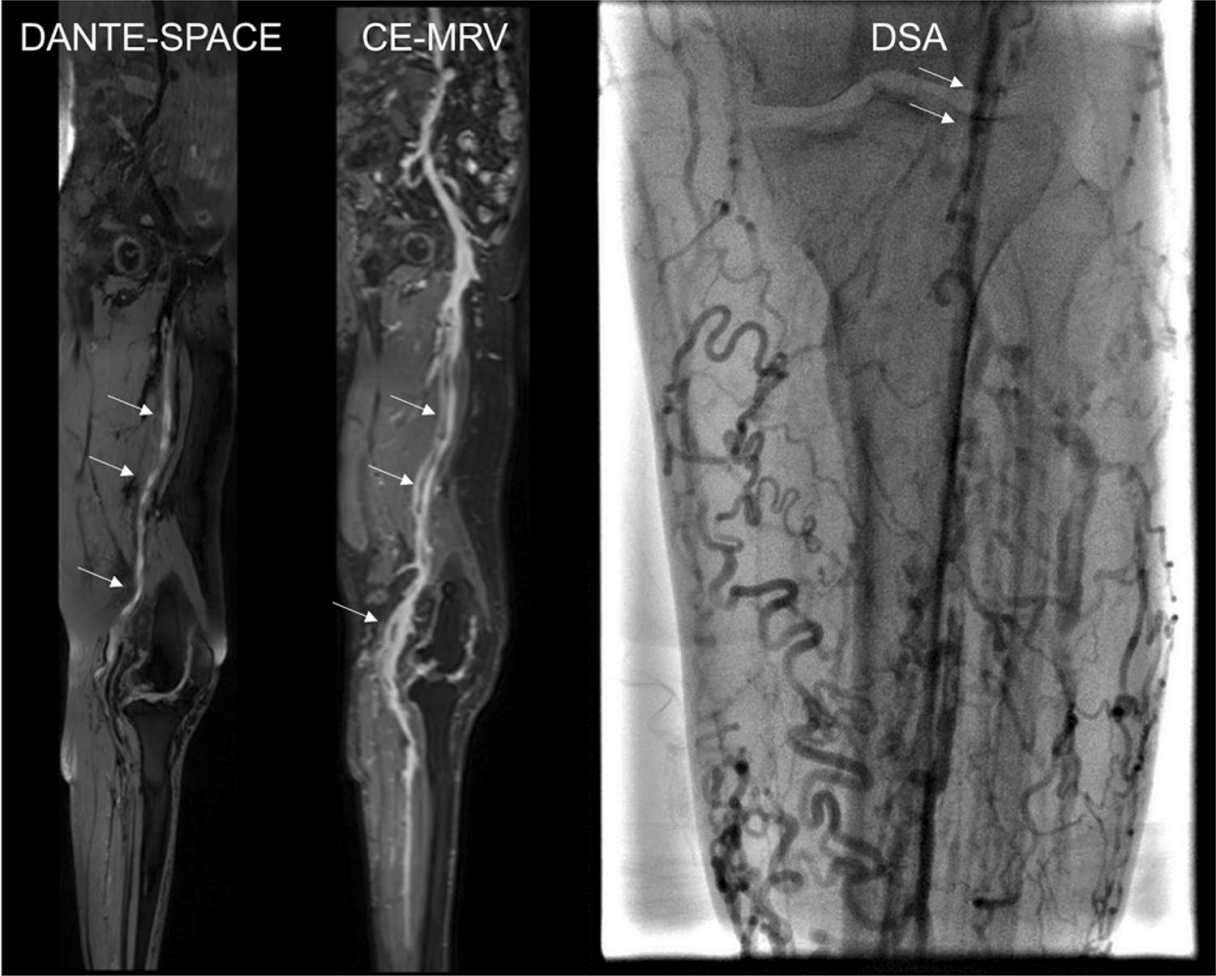
MRI and DSA images of an 85-year-old male with left lower extremity swelling and pain and superficial varicose veins. In the DANTE-SPACE images, the cavity of the common iliac and femoral veins was smooth and showed a low signal. There were multiple thrombi in the middle and lower femoral veins with a high signal at different degrees and rich signal layers, showing signs of a “flow void signal,” and the lesion in the venous vascular wall was rough. In the CE-MRV images, the common iliac and femoral veins were smooth and showed a high signal. In the middle and lower femoral veins, the thrombosis of the femoral vein showed multiple thromboses with a low signal that was characterized by a “filling defect.” The vein wall of the lesion was relatively smooth. In the DSA image, femoral and popliteal occlusion, collateral circulation, and superficial varicose veins were noted

**Fig. 2:**
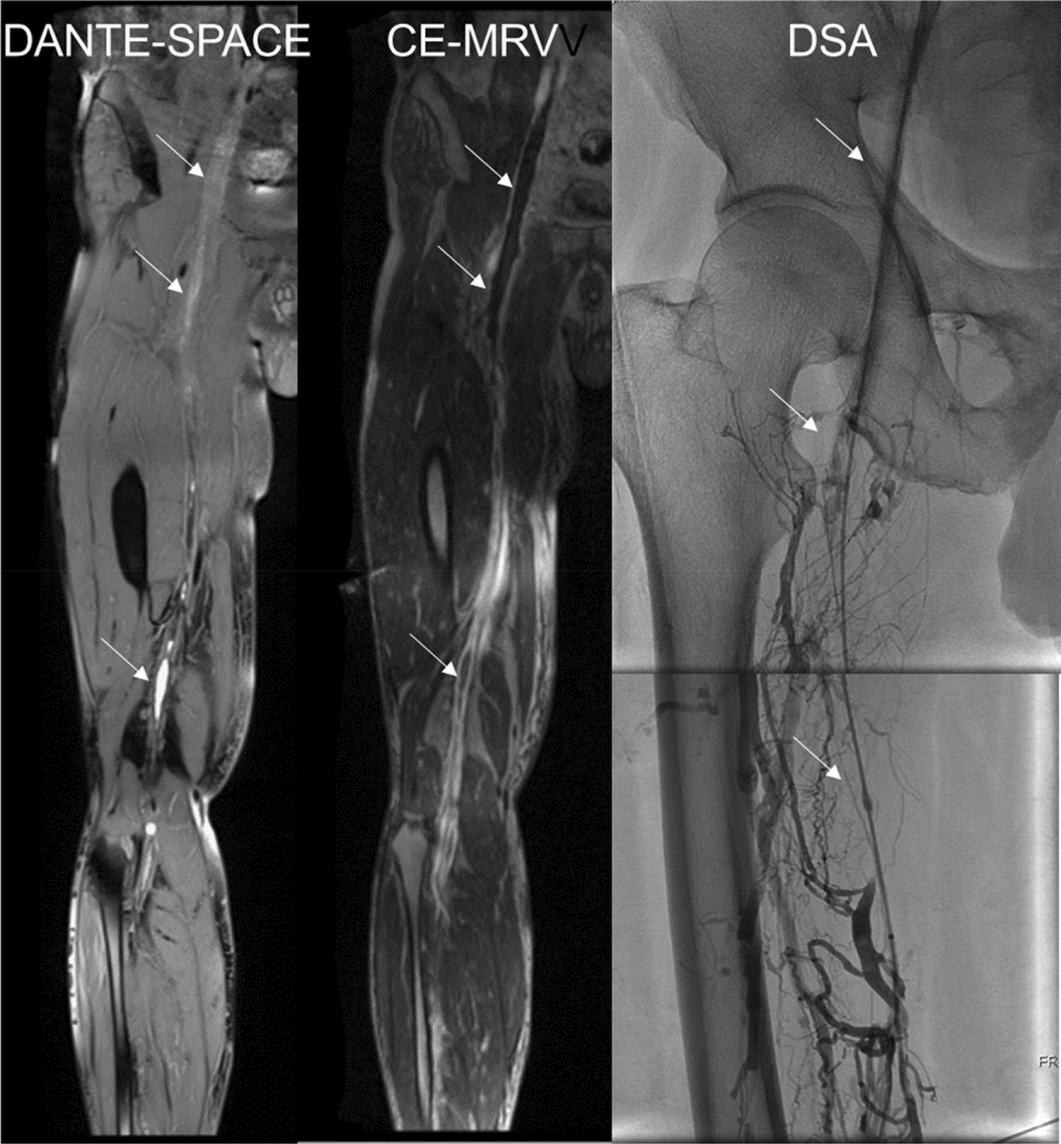
MRI and DSA images of a 61-year-old male with right lower extremity swelling and pain. In the DANTE-SPACE images, the venous thrombosis of the right lower extremity showed a heterogeneous high signal with a rich signal hierarchy and a rough vascular wall in the diseased segment. In the CE-MRV images, there were multiple thromboses in the right lower extremity veins. The thrombus showed a low signal “filling defect,” and the difference in the thrombus signal was not obvious. In the DSA images, there was a right lower extremity vein occlusion and collateral circulation formation. The right iliac and femoral vein proximal thrombosis was easy to dissolve, and contrast agent filled the cavity. The thrombosis in the middle femoral vein was not easy to dissolve; after the catheter was passed through the cavity, contrast agent still did not fill it

A total of 26 patients with DVT, comprising 14 women (one with an amputation of the right lower extremity) and 12 males, were enrolled. The average age was 60.3 ± 9.6 years (range, 43–85 years). The collected data comprised a total of 51 lower extremity veins, and 255 lower limb veins in the observed segments. The distribution of thrombus was obtained using different methods of examination (Tables 1, 2, and Figs. 1, 2). For the lower extremity deep venous thrombosis, the coincidence rate between DANTE-SPACE and conventional CE-MRV was 100% (n = 51), and the coincidence rate between DANTE-SPACE and DSA was also 100% (n = 51).

**Table 1:** Distribution of thrombus in DANTE-SPACE and CE-MRV image

Method/location	CIV	EIV	FV	PV	CV
DANTE-SPACE	14	18	23	21	18
CE-MRV	14	18	23	21	18

*CIV* common iliac vein, *EIV* external iliac vein, *FV* femoral vein, *PV* popliteal vein, *CV* calf vein

**Table 2:** Qualitative analysis of DVT

	CE-MRV		Total
	Negative	Positive	
DANTE-SPACE			
Negative	28	0	28
Positive	0	23	23
Total	28	23	51

The results showed that DANTE-SPACE and CE-MRV were consistent for the diagnosis of DVT (P < 0.05), and the consistency was good (Kappa = 1, P < 0.05)

In the DANTE-SPACE images, the unobstructed venous cavity of the lower extremities showed a low signal (flow void signal); the thrombus showed varying degrees of high signal and rich signal, as well as signs of “flow void interrupt,” and lesions in the vascular wall appeared to be rough and uneven. In the CE-MRV images, under the high-contrast agent, the unobstructed venous cavity of the lower extremity showed a high signal and the thrombus showed a low signal, characterized by a “filling defect.” The venous wall of the lesion was relatively smooth.

Regarding the magnetic resonance signal intensity ratio, the signal intensity of the thrombus/cavity ratio using DANTE-SPACE was significantly higher than that of CE-MRV (20.51 ± 12.96 vs. 0.51 ± 0.46; P < 0.05, n = 51), and the difference was statistically significant (Table 3). The signal intensity of the thrombus/muscle ratio using the DANTE-SPACE sequence was significantly higher than that found using the CE-MRV sequence (1.74 ± 0.57 vs. 0.99 ± 0.53; P < 0.05, n = 51). The difference was also statistically significant (Table 4).

**Table 3:** Quantitative analysis of the ratios of thrombus/cavity signal intensity

Groups	Mean ± SD	W value	*P* value
DANTE-SPACE	20.51 ± 12.96	− 4.541	< 0.05
CE-MRV	0.51 ± 0.46		

The results showed that the ratio of thrombus/cavity signal intensity measured by DANTE-SPACE was higher than that found with CE-MRV (P < 0.05)

**Table 4:** Quantitative analysis of the ratios of thrombus/muscle signal intensity

Groups	Mean ± SD	W value	*P* value
DANTE-SPACE	1.74 ± 0.70	− 3.484	< 0.05
CE-MRV	0.99 ± 0.53		

The ratio of thrombus/muscle signal intensity measured by DANTE-SPACE was higher than that found with the CE-MRV method (P < 0.05)

## Discussion

Ultrasound is widely used in the clinical diagnosis of deep venous thrombosis of the lower extremities. However, this imaging technique is more dependent on the personal experience and skills of the operating physician. The imaging field is small and easily leads to misdiagnosis of DVT. Ultrasound can only determine the distribution of the thrombus; there is great difficulty in judging the age or the source of the thrombosis. Compared with ultrasonography, CE-MRV has obvious advantages for the diagnosis and analysis of lower extremity deep venous thrombosis [4, 5]. However, magnetic resonance contrast-enhanced scanning requires the use of gadolinium-containing contrast agent. As early as 2006, the U.S. Food and Drug Administration (FDA) released to the public a health monitoring report demonstrating that nephrogenic systemic fibrosis (NSF) and nephrogenic fibrosing dermopathy (NFD) are associated with gadolinium-containing contrast agents used by patients during an enhanced MRI scan or CE-MRA. RJ McDonald et al. [6] found that gadolinium deposition in neural tissues after GBCA administration occurs in the absence of intracranial abnormalities that might affect the permeability of the blood–brain barrier. These findings challenge the current understanding of the biodistribution of these contrast agents and their safety. Therefore, the use of non-enhanced MRI diagnosis of DVT will be the basis of future development of this method.

Karla Maria Treitl et al. [7] conducted a comparative study of 13 patients with DVT (11 cases were confirmed by ultrasound and 2 were suspected due to the presence of pulmonary embolism) on a 3.0T magnetic resonance scanner. As a result, VISTA-MRI and CE-MRI image quality and diagnostic confidence were as follows: 3.54 vs. 3.55 and 3.80 vs. 3.77, respectively (P < 0.001). There was high consistency between VISTA and CE-MRI in the diagnosis of DVT based on CE-MRI. VISTA (some companies call this method SPACE) has the advantages of a high signal-to-noise ratio, fast imaging speed, and natural flow signal suppression. However, its suppression of blood flow signals, which are generally used for arterial angiography with faster blood flow, is quite limited [8]. Due to very slow venous flow, VISTA cannot completely suppress the blood flow signal in the venous cavity, making imaging ineffective. It is difficult to distinguish the vessel wall, blood flow, and thrombus, and it is therefore difficult to assess the age or source of the thrombus.

The DANTE-SPACE sequence was prepared with a blood flow-suppressing pulsed DANTE, and the data were read using SPACE. DANTE is sensitive to motion signal, and SPACE also has a blood flow signal suppression effect. Therefore, DANTE -SPACE can well suppress slow-flowing blood flow signals, clearly showing the non-flowing components in the lumen and the state of the venous blood vessels, which will facilitate the detailed observation of venous thrombosis [9]. Figures 1 and 2 show DANTE-SPACE images in which normal venous blood flow shows a significant low signal (black blood technique), venous thrombosis shows varying degrees of hyperintense, and the diseased segment vein wall is rough.

CE-MRV needs enhanced contrast agents for its operation. Under the contrast agent, the normal blood flow in the endovascular cavity is bright and has a high signal (white). In general, it is difficult for the contrast agent to enter the thrombus, which is obviously low signal (black). Therefore, “intracavity filling defect” and “blood flow interruption” are important signs of conventional CE-MRI for the diagnosis of deep venous thrombosis in the lower extremities. DANTE-SPACE uses black-blood technology, it is contrast-free, and it shows markedly low signal (black) in blood flowing normally in the vessel bottom, whereas hemoglobin and collagen fibers in different oxidized states inside the thrombus show different levels of high signal (different levels of white). Thus, “abnormally high intracavity signal” and “interrupted flow void” phenomena are important signs for the DANTE-SPACE diagnosis of deep venous thrombosis.

In this experiment, the thrombus/cavity signal intensity ratios in the DANTE-SPACE and CE-MRV images were as follows: 20.5 ± 12.96 vs. 0.51 ± 0.46, n = 51 (P < 0.05); and the ratios of thrombus/muscle signal intensity were as follows: 1.74 ± 0.70 vs. 0.99 ± 0.53, n = 51 (P < 0.05). In DANTE-SPACE, thrombus and cavity, and thrombus and surrounding muscle tissue have a stronger signal contrast. Relatively high-signal thrombus lesions are more likely to draw the attention of the diagnostician, who is more apt to then accurately diagnose the presence and distribution of intravenous thrombosis given evidence of a bright lesion on a dark background.

In the past, there were three stages of deep venous thrombosis in the lower extremities, based on the different times of onset of the disease. In the acute period, the onset time is less than two weeks; in the subacute period, onset time is between two weeks and six months; and in the chronic period, the onset time is more than six months. This staged method is primarily based on clinical symptoms and ignores the actual process of thrombosis; therefore, it is more likely that staging and clinical efficacy cannot be used to verify one another. Phinikaridou et al. [10] believed that magnetic resonance motion imaging and diffusion-weighted imaging can identify the protein components of the thrombus and delineate the different stages of thrombosis.

As normal flowing blood coagulates, hemoglobin during coagulation undergoes a series of characteristic oxidative changes over time and exhibits different MRI signal characteristics. In the acute phase, deoxygenated hemoglobin is primarily localized to the cells, and T1WI has an equal signal. In subacute coagulation, erythrocyte disintegration occurs, methemoglobin containing Fe3 + is paramagnetic, and T1WI has a high signal. In the chronic phase, hemoglobin disintegration and hemosiderin deposition occur, and T1WI has a slightly lower signal. The different states of hemoglobin render it like an endogenous contrast agent, suggesting different “ages” of the thrombus [11–13]. In addition, Prakash Saha et al. [14] established a mouse DVT model and found that there was a corresponding relationship between the T1 relaxation time and thrombus age in the venous thrombus, with the longest T1 time in the super acute phase and the shortest T1 time in the subacute phase. The T1 time of the chronic stage gradually became longer and closer to the T1 value of normal blood.

When acute thrombosis of the lower extremities occurs, it often leads to thrombophlebitis. An acute inflammatory reaction can cause capillary leakage in the corresponding area. In the conventional CE-MRV sequence, capillary leakage will cause an increase in static contrast agent extravasation, manifested as tissue around the vein thrombus strengthening, showing a high signal, with the thrombus showing a relatively low signal, a pattern comprising the “bull’s eye” sign [5]. This sign can be enhanced or decreased with a reduction of the inflammatory response and can also be used as a basis for the assessment of acute venous thrombosis by CE-MRV. In this respect, because DANTE-SPACE does not use contrast agent, the display of pre-thrombotic phlebitis is less obvious than that seen with CE-MRV. However, compared with conventional CE-MRV, DANTE-SPACE has the same ability to detect venous thrombosis in the lower extremity.

In my experiment, DANTE-SPACE had some limitations. (1) DANTE-SPACE takes 4 min to complete a segment scan of the lower limb, and it takes about 12 min to cover the whole lower limb. Compared with CE-MRI, DANTE-SPACE had a longer scanning time and a higher specific absorption rate. (2) DANTE-SPACE technology requires high uniformity of magnetic field (B0). In the center area of the image, the magnetic field uniformity is good and the image quality is good; in the edge area of the image, the magnetic field uniformity is poor and the image quality is easy to deteriorate. (3) Poor display of small diameter veins in the calf.

This study had several limitations. First, the sample size was small, and more patients are needed to further prove the experimental conclusions drawn. Second, there was no comparison for the clinical effect, and the value of guiding the clinical treatment needs further study.

## Conclusions

The DANTE-SPACE technique shows promise as an effective diagnostic method for DVT, particularly for patients who are not suitable for or willing to use gadolinium-enhanced contrast agents. DANTE-SPACE images can show early abnormal expression in the venous wall and display venous thrombus with abundant signal levels. Further studies should be conducted to reveal the relationship between the signal enhancement difference and the thrombus age.
